# ELR(+) chemokine signaling in host defense and disease in a viral model of central nervous system disease

**DOI:** 10.3389/fncel.2014.00165

**Published:** 2014-06-17

**Authors:** Martin P. Hosking, Thomas E. Lane

**Affiliations:** ^1^Department of Molecular Biology and Biochemistry, University of CaliforniaIrvine, CA, USA; ^2^Department of Pathology, Division of Microbiology and Immunology, School of Medicine, University of UtahSalt Lake City, UT, USA

**Keywords:** chemokines, chemokine receptors, virus, neuroinflammation, demyelination

## Abstract

Intracranial infection of the neurotropic JHM strain of mouse hepatitis virus (JHMV) into the central nervous system (CNS) of susceptible strains of mice results in an acute encephalomyelitis, accompanied by viral replication in glial cells and robust infiltration of virus-specific T cells that contribute to host defense through cytokine secretion and cytolytic activity. Mice surviving the acute stage of disease develop an immune-mediated demyelinating disease, characterized by viral persistence in white matter tracts and a chronic neuroinflammatory response dominated by T cells and macrophages. Chemokines and their corresponding chemokine receptors are dynamically expressed throughout viral infection of the CNS, influencing neuroinflammation by regulating immune cell infltration and glial biology. This review is focused upon the pleiotropic chemokine receptor CXCR2 and its effects upon neutrophils and oligodendrocytes during JHMV infection and a number of other models of CNS inflammation.

## Introduction

Intracranial infection of susceptible mice with the JHM strain of mouse hepatitis virus (JHMV) causes an acute encephalomyelitis followed by a chronic demyelinating disease. JHMV, after initially infecting ependymal cells lining the ventricles, rapidly disseminates to astrocytes, oligodendroglia, and microglia throughout the brain and spinal cord (Wang et al., 1992). Although inflammatory virus-specific T cells are efficient in controlling viral replication through the secretion of IFN-γ and cytolytic activity, sterile immunity is not achieved. Viral protein and/or RNA persist within oligodendroglia and drive continual T cell and macrophage infiltration, leading to chronic neuroinflammation and demyelination. Histological features associated with viral persistence include the development of an immune-mediated demyelinating disease similar to the human demyelinating disease MS; both T cells and macrophages are critical mediators of disease severity, contributing to myelin damage (Cheever et al., 1949; Perlman et al., 1999).

Through the course of acute and chronic JHMV-induced neurologic infection, there is a coordinated expression of chemokines and chemokine receptors that regulate inflammation, contributing to both host defense and disease exacerbation. Among the chemokines expressed during infection are members of the ELR(+) chemokine family CXCL1 and CXCL2. CXCL1 and CXCL2 are potent chemoattractants for peripheral mononuclear cells (PMNs), binding and signaling through their receptor CXCR2 (Wolpe et al., 1989; Moser et al., 1990; Schumacher et al., 1992; Marro et al., 2012; Weinger et al., 2013). Moreover, PMNs have been shown to enhance central nervous system (CNS) inflammation by disrupting blood brain barrier (BBB) integrity in animal models of spinal cord injury (SCI; Tonai et al., 2001; Gorio et al., 2007), autoimmune demyelination (Carlson et al., 2008), and JHMV-induced encephalomyelitis (Zhou et al., 2003), while blocking or silencing of CXCR2 signaling mutes inflammation and tissue damage in mouse models in which PMN infiltration is critical to disease initiation (Kielian et al., 2001; Belperio et al., 2005; Londhe et al., 2005a,b; Strieter et al., 2005; Gorio et al., 2007; Wareing et al., 2007; Carlson et al., 2008).

CXCR2 is also expressed by oligodendrocytes (Omari et al., 2005), and CXCL1 promotes the proliferation and positional migration of oligodendrocyte precursor cells (Robinson et al., 1998; Robinson and Franic, 2001; Tsai et al., 2002; Filipovic and Zecevic, 2008). Further, both CXCR2 and CXCL1 are expressed within active MS lesions (Omari et al., 2005, 2006). How and whether CXCR2 and its cognate ligands regulate immune and glial cell function during acute and chronic disease of the CNS is the focus of this review.

## ELR(+) chemokine signaling promotes PMN infiltration into the CNS during acute JHMV infection

**Figure 1:**
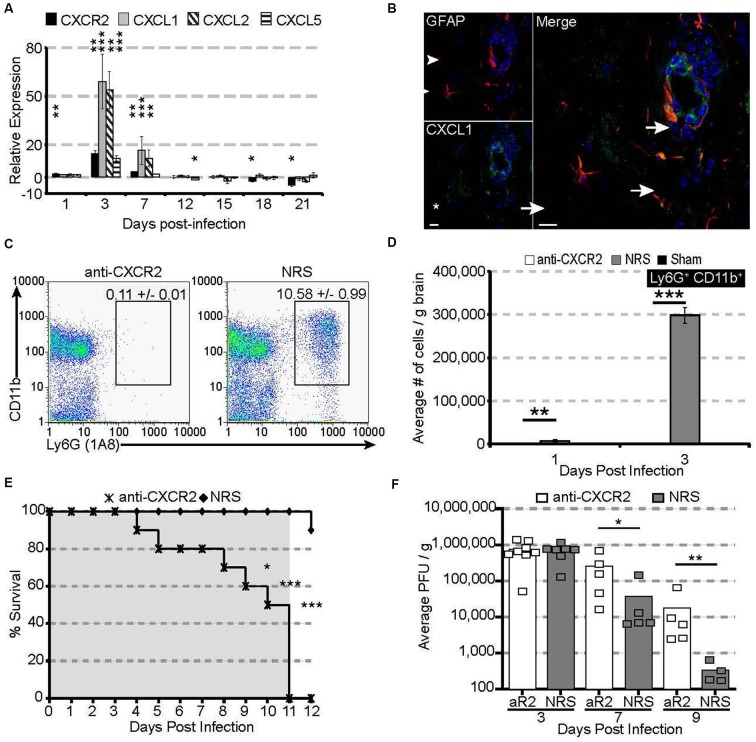
**CXCR2 drives neutrophil infiltration into the CNS during acute JHMV infection**. C57BL/6 mice were infected with JHMV and their brains removed at the indicated time points. **(A)** mRNA for CXCR2, CXCL1, and CXCL2 are upregulated within the brains of JHMV infected mice. **(B)** Immunofluorescence staining reveals that the majority of CXCL1 (green) co-localizes with GFAP+ (red) astrocytes. **(C)** Representative FACS plots depicting the average frequency of neutrophils at day 3 are shown in panel. **(D)** Neutralization of CXCR2 blocks neutrophil (Ly6G^+^CD11b^+^) infiltration into the CNS. **(E)** CXCR2 neutralization results in 100% morality by day 11 pi (shaded area indicates the treatment period) and **(F)** elevated viral loads within the brains of treated mice. *NRS* = normal rabbit serum treated mice. * *p* < 0.05, ** *p* < 0.01, *** *p* < 0.001 compared to NRS-treated mice.

Following JHMV infection, mRNA for the chemokine receptor CXCR2 and its associated ligands CXCL1 and CXCL2 are significantly upregulated within the acutely infected CNS, peaking at 3 days pi (Figure 1A). CXCL1 expression was localized to astrocytes (GFAP-positive) within the parenchyma and associated with the microvasculature (Figure 1B), consistent with previous observations (Lane et al., 1998; Omari et al., 2006; Rubio and Sanz-Rodriguez, 2007). The expression of the CXCR2 ligands within the CNS closely paralleled neutrophil emergency release into the circulation and infiltration into the CNS; CXCR2-expressing neutrophils were detectable as early as 1 day pi and peaked at 3 days pi within both the periphery and the CNS (Hosking et al., 2009).

To determine whether CXCR2—signaling controlled neutrophil infiltration into the CNS, JHMV-infected mice were treated with either CXCR2 antiserum or control serum (NRS). Neutralization of CXCR2 almost completely abrogated neutrophil infiltration into the CNS (Figures 1C,D). Without infiltrating neutrophils, permeabilization of the blood-brain barrier was impaired (Hosking et al., 2009) and subsequent inflammatory cell infiltration was significantly reduced. Mice treated with CXCR2 neutralizing antiserum were incapable of controlling viral replication, and 100% of all infected mice succumbed to viral infection within 11 days and this was associated with an impaired ability to control CNS viral replication (Figures 1E,F). Moreover, total and virus specific CD4^+^ and CD8^+^ T cell infiltration into the CNS was diminished. Notably, CXCR2 neutralization did not alter the peripheral generation of virus-specific T cells, indicating that the increased mortality and diminished ability to control viral infection within the CNS is likely associated with the dampened access of T cells into the CNS parenchyma (Hosking et al., 2009). Collectively, these data demonstrate that during viral infection of the CNS, CXCR2 and its associated chemokines function to non-redundantly attract neutrophils into the CNS, where they are required to permeabilize the blood-brain barrier, thus facilitating subsequent inflammatory cell infiltration and control of viral replication.

## ELR(+) chemokine signaling and neutrophils in other models of CNS inflammation

Neutrophils are amongst the earliest inflammatory infiltrate into the CNS following experimental autoimmune encephalitis (EAE) induction, and their presence precedes axonal damage, demyelination, and clinical disease (Carlson et al., 2008; Soulika et al., 2009; Wu et al., 2010). Neutralization of either CXCR2 (Carlson et al., 2008) or CXCL1 (Roy et al., 2012) potently reduces neutrophil infiltration into the CNS and reduces BBB permeability, thereby significantly delaying the onset and peak of clinical symptoms. Neutrophils also infiltrate into the CNS during the first week following cuprizone feeding, and their early presence in the CNS is absolutely necessary for the subsequent demyelination observed within the corpus callosum (Liu et al., 2010a). CXCR2 deficient mice or bone marrow chimeric mice, where myeloid cells lack CXCR2, or neutrophil-depleted mice are resistant to cuprizone induced demyelination (Liu et al., 2010a). Interestingly, although neutrophils are also critical for lymphocytic choriomeningitis virus (LCMV)- and pilocarpine-induced BBB permeabilization and subsequent seizures (Fabene et al., 2008; Kim et al., 2009), they are dispensable for seizures during Theiler’s murine encephalomyelitis virus (TMEV; Libbey et al., 2011), underlining the fact that neutrophils are not the only cell type capable of mediating permeabilizing the BBB. To this point, resident monocytes, astrocytes, and CD8^+^ T cells are all capable of direct permeabilization (Savarin et al., 2010, 2011; Johnson et al., 2012). Nevertheless, CXCR2-directed neutrophil infiltration into the CNS is a key determinate for subsequent inflammatory cell infiltration in a variety of CNS models of viral infection, demyelination, and autoimmunity.

## ELR(+) chemokine signaling promotes oligodendroglia survival during chronic JHMV-induced demyelination

How chemokine receptor signaling contributes to chronic neurologic diseases has largely been considered within the context of targeted leukocyte recruitment into the CNS (Liu et al., 2000, 2001a,b; Glass and Lane, 2003; Hosking et al., 2009). However, numerous resident cell types of the CNS also express chemokine receptors under non-inflammatory and inflammatory conditions (reviewed in Bajetto et al., 2001; Ubogu et al., 2006), indicating that these cells are capable of responding to specific chemokine ligands. Thus, chemokine signaling may participate in either repair and/or exacerbation of pathology following insult, injury, or infection of the CNS (Liu et al., 2001b; Kerstetter et al., 2009; Omari et al., 2009).

**Figure 2:**
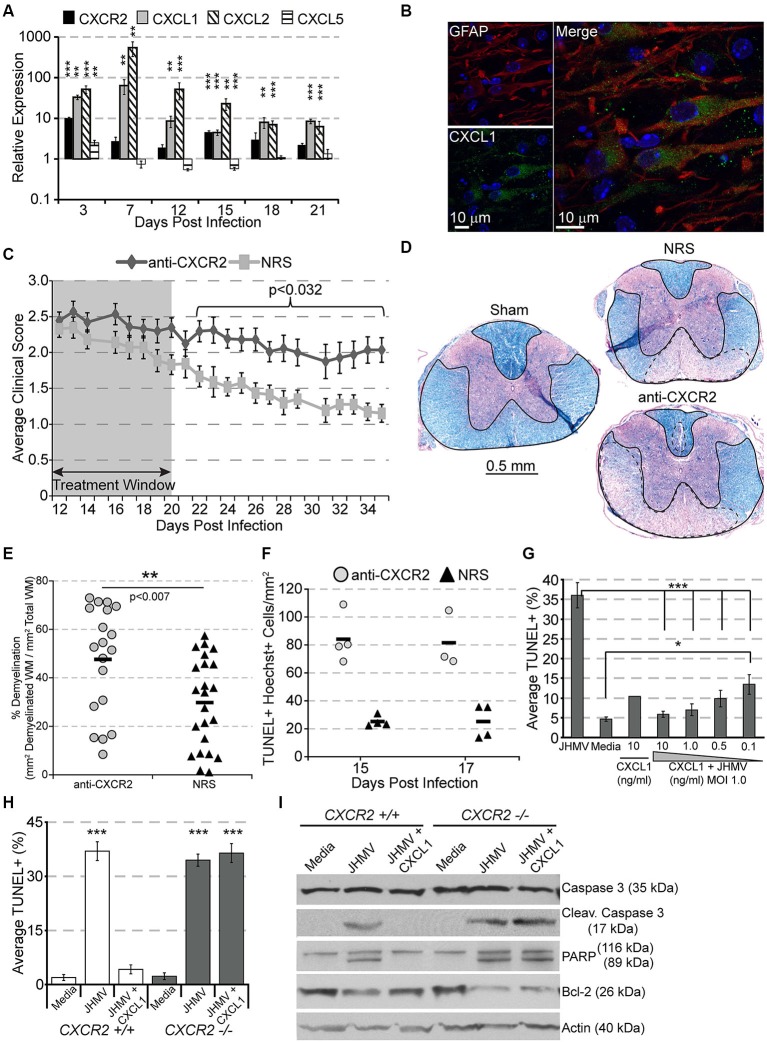
**CXCR2 promotes spontaneous recovery and oligodendrocyte survival during chronic JHMV infection**. C57BL/6 mice were infected with JHMV and their spinal cords removed at the indicated time points. **(A)** mRNA for CXCR2, CXCL1, and CXCL2 are upregulated within the spinal cords of JHMV infected mice. **(B)** Immunofluorescence staining reveals that the majority of CXCL1 (green) co-localizes with GFAP-positive (red) astrocytes within the spinal cord white matter. **(C)** Neutralization of CXCR2 (from day 12–20 pi) delays clinical recovery from chronic JHMV infection. **(D and E)** Mice receiving CXCR2 antiserum had significantly greater total areas of demyelination within the spinal cord. Representative luxol fast blue stained spinal cords are shown in panel **(D)** with the total (solid line) and demyelinated (dashed line) white matter indicated. **(F)** Significantly (*p* < 0.001) increased numbers of apoptotic (TUNEL+) cells were observed within the spinal cords of anti-CXCR2 treated mice. **(G)** CXCL1, in a dose-dependent manner, protects oligodendrocytes from apoptosis, and **(H)** CXCR2-deficienct oligodendrocyte-enriched cultures are not protected from apoptosis. **(I)** Protein lysates from CXCR2-sufficient and CXCR2-deficient oligodendrocyte cultures were assessed via western blot for total caspase 3, activated caspase 3, PARP, Bcl-2, and actin expression.* NRS* = normal rabbit serum treated mice. * *p* < 0.05, ** *p* < 0.01, *** *p* < 0.001 compared to NRS-treated mice.

Following JHMV infection, mRNA transcripts for CXCR2 as well as its ligands CXCL1 and CXCL2 are significantly upregulated, persisting until at least 21 days pi within the spinal cord (Figure 2A). CXCL1 expression was localized to GFAP+ astrocytes within the white matter (Figure 2B), suggesting that CXCR2, besides attracting neutrophils during early acute viral infection, may also alternatively function during chronic demyelination. To determine whether CXCR2 signaling was beneficial or pathogenic, mice persistently infected with JHMV were treated with anti-CXCR2 or control serum (NRS) from day 12–20 p.i. CXCR2 neutralization significantly delayed spontaneous clinical recovery (Figure 2C). Correspondingly, spinal cords from anti-CXCR2 treated mice revealed significantly greater areas of demyelination (Figures 2D,E). Importantly, CXCR2 neutralization during chronic JHMV infection did not affect inflammatory cell infiltration into the CNS (Hosking et al., 2010).

CXCR2 neutralization was also associated with an increase of apoptotic oligodendrocytes and oligodendrocyte precursor cells within white matter tracts of the spinal cord (Figure 2F; Hosking et al., 2010). To determine whether or not CXCR2 could directly prevent JHMV-mediated apoptosis, cultured oligodendroglia were infected with JHMV *in vitro* and treated with varying concentrations of CXCL1. In accordance with previous observations (Liu et al., 2003, 2006; Liu and Zhang, 2005, 2007), JHMV—infected oligodendrocytes readily underwent apoptosis (Figure 2G), and western blotting confirmed activated caspase 3, cleaved poly ADP ribose polymerase (PARP) (a caspase 3 target), and muted expression of Bcl-2 (Figure 2I). CXCL1, in a dose-dependent manner, prevented JHMV-mediated apoptosis (Figure 2G). Moreover, activated caspase 3 and cleaved PARP were undetectable in CXCL1-treated cultures (Figure 2I). Notably, CXCL1 was incapable of rescuing CXCR2 deficient cultures from JHMV-mediated apoptosis (Figures 2H,I). CXCR2 also prevents IFNγ-and CXCL10- mediated apoptosis of murine or human oligodendroglia cultures (Tirotta et al., 2011, 2012). Collectively, these data suggest that CXCR2, during chronic viral infection of the CNS, prevents oligodendrocyte apoptosis and promotes clinical recovery from viral induced demyelination.

## ELR(+) chemokine signaling and other models of CNS inflammation and demyelination

The role for CXCR2 signaling during EAE and a variety of toxin—induced demyelination models has also been studied. Raine and colleagues (Omari et al., 2009) have shown that CXCL1, when inducibly expressed by astrocytes after the onset of EAE, reduces peak disease severity, reduces total demyelination, and increases the onset of remyelination. Moreover, transgenic CXCL1 was associated with greater proliferation (presumably of oligodendrocyte precursors) throughout the spinal cord white matter (Omari et al., 2009). Conversely, Ransohoff and colleagues (Liu et al., 2010b) have demonstrated, using a series of bone marrow chimeras, that parenchymal CXCR2 deficiency on radio-resistant cells promotes faster recovery from EAE, cuprizone—induced demyelination, and *in vitro* lysotecithin-induced demyelination. Notably, initial clinical severity, inflammation, and/or demyelination in all three models of demyelination and repair were similar regardless of whether parenchymal cells possessed CXCR2; accelerated recovery was associated with initial increases in oligodendrocyte precursor cells, followed by an increased density of mature myelinating oligodendrocytes (Liu et al., 2010b). Similar results were observed following CXCR2 chemical anatagonism during EAE and *in vivo* lysolecithin-induced demyelination (Kerstetter et al., 2009).

## Perspectives

The JHMV-induced model of viral-induced encephalomyelitis provides an important tool in defining molecular and cellular mechanisms that regulate neuroinflammation during both host defense and disease progression. Our research on chemokines and chemokine receptors has revealed important roles for these molecules in orchestrating CNS inflammation in response to JHMV infection. We and others have found unique and pleiotropic roles for ELR+ chemokine signaling via CXCR2 in moderating neutrophil infiltration and protecting oligodendroglia from apoptosis in response to exposure to virus and proinflammatory cytokines. Ongoing research in our laboratory continues to focus on the role of ELR(+) chemokine signaling on oligodendroglia during JHMV-induced neuroinflammation. It will be important to analyze the effects of selectively ablating CXCR2 on oligodendroglia during JHMV-induced demyelination, while simultaneously manipulating the cellular sources of ELR-positive chemokines in the CNS that may promote neuroprotection during chronic JHMV-induced disease.

## Conflict of interest statement

The authors declare that the research was conducted in the absence of any commercial or financial relationships that could be construed as a potential conflict of interest.
